# Ketogenic Diet for Obese COVID-19 Patients: Is Respiratory Disease a Contraindication? A Narrative Review of the Literature on Ketogenic Diet and Respiratory Function

**DOI:** 10.3389/fnut.2021.771047

**Published:** 2021-12-09

**Authors:** Elena Gangitano, Rossella Tozzi, Stefania Mariani, Andrea Lenzi, Lucio Gnessi, Carla Lubrano

**Affiliations:** ^1^Department of Experimental Medicine, Sapienza University of Rome, Rome, Italy; ^2^Department of Molecular Medicine, Sapienza University of Rome, Rome, Italy

**Keywords:** SARS-CoV-2, COVID-19, ketogenic diet, low-carbohydrate high-fat diet, obesity, VLCKD, respiratory disease, respiratory failure

## Abstract

Morbid obese people are more likely to contract SARS-CoV-2 infection and its most severe complications, as need for mechanical ventilation. Ketogenic Diet (KD) is able to induce a fast weight loss preserving lean mass and is particularly interesting as a preventive measure in obese patients. Moreover, KD has anti-inflammatory and immune-modulating properties, which may help in preventing the cytokine storm in infected patients. Respiratory failure is actually considered a contraindication for VLCKD, a very-low calorie form of KD, but in the literature there are some data reporting beneficial effects on respiratory parameters from ketogenic and low-carbohydrate high-fat diets. KD may be helpful in reducing ventilatory requirements in respiratory patients, so it should be considered in specifically addressed clinical trials as an adjuvant therapy for obese patients infected with SARS-CoV-2.

## Introduction

### Obesity and COVID-19: An Emergency in the Emergency

The pandemic of SARS-CoV-2 infection has been challenging the world for over a year. Many risk factors for the development of Coronavirus 2019 disease (COVID-19) have been identified, and among these, metabolic diseases play a major role. Severe obesity is associated with a greater risk of severe COVID-19 (1, 2), ICU admission (1) and need for invasive mechanical ventilation (2, 3). Moreover, obesity may be a risk factor for developing COVID-19 at a younger age (4).

The current pandemic, with lockdown imposed to reduce the spread of the virus, is being leading to social distancing and long times at homes. The reduced possibility of exercising outdoor and the reduced spontaneous outdoor activity, the closure of the gyms and the swimming pools, together with the increase of stress-related disorders, may lead lots of people to a worsening of the weight excess and related comorbidities.

In the real-world setting, health professionals face many practical difficulties in treating obese patients. The excess of adipose tissue hinders the diagnosis with pulmonary ultrasound, delaying the intervention in advanced stages, with consequent higher mortality (5). In addition, the facilities for severely obese patients in the wards and intensive care units are lacking, so that interventional procedures as intubation may be slow and exert a negative impact on the prognosis of the patients (5). Moreover, obese people develop a reduced response to vaccinations (6) and central obesity has been recently associated with lower antibody titres in response to COVID-19 mRNA vaccine (7). Therefore, strategies aimed to reduce weight excess are mandatory.

### Ketogenic Diet

Ketogenic diet is a dietary approach characterized by the consumption of a very low amount of carbohydrates, <50 g/day, with consequent development of ketosis. Fat is used as a primary source of energy, through the beta-oxidation of fatty acids. There are different kinds of ketogenic diets, defined on the basis of the macronutrient composition.

The low-carbohydrate high-fat ketogenic diet (LCHF) is characterized by the absence of a limit for calorie intake and fat intake, which is around 80–90% of total day energy. The low-calorie diet (LCD) provides among 800–1,200 Kcal/day and the very low-calorie diet (VLCD) is characterized by an even more strict calorie restriction (<800 Kcal/day), but they do not necessary lead to ketosis. The very-low calorie ketogenic diet (VLCKD) is characterized by a similar caloric restriction, but is always associated to ketosis, and is particularly interesting for the treatment of obesity and its comorbidities. In 2019 the Italian Society of Endocrinology released a consensus statement on the administration of VLCKD for the management of metabolic diseases (8), and respiratory failure was counted among the absolute contraindications. Anyhow, the use of KDs is recently spreading in new proposed fields of application, and some pathological features which are currently considered contraindications may benefit from its tailored use on the single patient, by experienced physicians (9).

The prescription of KD is currently under consideration in other pathologies than obesity, as headache (10), polycystic ovary syndrome (11), cancer (12), and neurodegenerative diseases (13, 14).

### Ketogenic Diet and COVID-19

**Figure 1:**
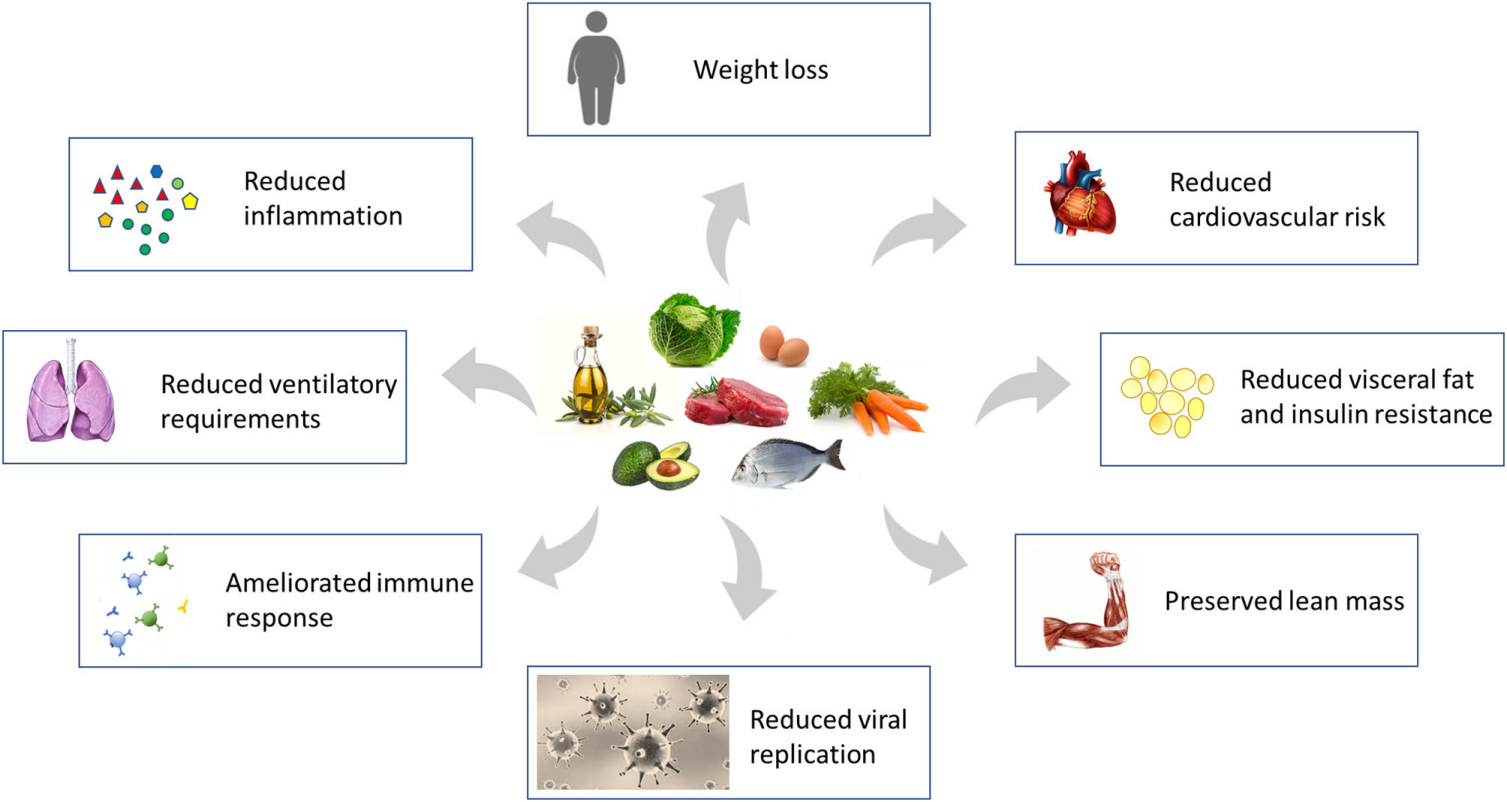
Mechanisms through which Ketogenic Diet directly, and its consequent weight loss indirectly, may reduce the susceptibility to severe SARS-CoV-2 infection and stem the damage induced by the virus. Modified from Gangitano et al. (22).

KD may be helpful in fighting COVID-19 through many mechanisms (see Figure 1). Severe SARS-CoV-2 infection determines a large innate immune response and ineffective adaptive immune response, that in some patients are associated with a cytokine storm and acute respiratory distress syndrome (15, 16). In fact, a virus infection with cytokine storm leads to an increase of reactive oxygen species and nitrogen species, which downregulate or inactivate many metabolic enzymes. Therefore, B and T cell proliferation is impaired, and cytokine production and cell death increase. These features of severe infections are related to reduced energy metabolism, altered redox state, oxidative damage and cell death, and may be at least partially dammed by KD. Ketone bodies have anti-inflammatory properties, since they are able to inhibit the NLRP3 inflammasome (17–19) and histone deacetylases (20), and consequently KD may reduce the risk of developing the cytokine storm, which is counted among the worst pathological features of COVID-19 (16, 21).

KD may be able to reduce viral replication and assembly (23), because many viruses, as varicella-zoster virus (24), hepatitis C (25) and cytomegalovirus (26), are dependent on fatty acid metabolism pathway for their replication cycle, and fatty acid synthesis is usually reduced in KD, thanks to the metabolic switch induced by the diet. The metabolic switch in the liver consists in passing from the glucogenic/glycolytic pathway (fed state) to the ketogenic pathway (fast state) (23).

Thanks to these antiviral and anti-inflammatory properties, Soliman et al. proposed the use of KD and intermittent fasting as a prophylactic and adjuvant therapy for SARS-CoV-2 infection (23). KD administration has also been hypothesized as a preventive measure in obese patients, in order to achieve a fast weight loss preserving lean mass, and a supportive care for obese COVID-19 patients (22, 27).

A recent animal model study with aged mice infected with a natural murine beta coronavirus observed that the ketogenesis led to an expansion of tissue protective γδ T cells, deactivation of NLRP3 inflammasome and remodeling of inflammatory monocytes in the lungs (28).

KD administration during lockdown may rise some concerns regarding the monitoring of patients. Anyway, KD has been administered to young patients and the studies showed good results in terms of safety and maintenance of ketosis. HFKD or modified Atkins diet (MAD; macronutrient ratio fat/carbohydrates+proteins usually of 1:1, and carbohydrates limited to 10–20 g/day) have been administered to children and young patients with uncontrolled seizures, and the authors nor the members of the International Ketogenic Diet Study Group, pediatric consensus group, reported any issues (34). Patients were followed with a mixed approach of in person meetings and telemedicine visits. Similarly, another study reported no major issues using teleassistance for the maintenance of patients on KD (34). Most parents of drug-resistant epilepsy pediatric patients were satisfied of the telemedicine approach (35, 36) and would recommend it regardless of the pandemic (36).

The administration of KD in infected patients is under evaluation in ongoing clinical trials, and the effects on respiratory function have not been clearly evaluated yet. A retrospective study showed a possible beneficial effect of an eucaloric ketogenic diet (carbohydrates <30 g/day) in COVID-19 patients admitted to hospital, studying a sample of 34 patients compared with 68 patients receiving an isocaloric standard diet, but the authors did not assess respiratory function, except for the need for CPAP (Continuous Positive Airway Pressure), which did not differ among groups (37).

The aim of our study is to review the literature on ketogenic diets in respiratory patients, to consider the possibility of their safe use, from a respiratory point of view, in COVID-19 obese patients.

## Low-Carbohydrate High-Fat Diets and Respiratory Function in Healthy Subjects

On a pathophysiological basis, fat oxidation produces less carbon dioxide (CO_2_) per amount of oxygen consumed compared to carbohydrates (CHO) (38), resulting in reduced ventilatory requirements. Therefore, ketogenic diet decreases metabolic carbon dioxide production for a given oxygen consumption, and may theoretically lead to a reduction in arterial carbon dioxide partial pressure (PETCO_2_), pulmonary ventilation, and CO_2_ body stores (29, 30).

Carbohydrates loads have been reported to precipitate respiratory failure in patients with lung compromise (39–42) since they may worse respiratory acidosis in patients unable to improve ventilation as a compensatory mechanism to excrete more CO_2_ (39, 43).

Over time, some authors investigated the effects of low-carbohydrate high-fat diets or supplements on respiratory parameters in healthy subjects, and most of them reported beneficial effects (see Table 1). In the majority of studies we report below, patients were administered an amount of CHO that could possibly lead to ketosis, but the development of ketosis wasn't verified.

**Table 1:** Summary table of the interventional studies on the effects of low-carbohydrate dietary interventions (minimum 5 days of intervention) on ventilatory parameters and pulmonary function in spontaneously breathing patients.

**References**	**Patients**	**Diet composition**	**Length of the dietary intervention**	**Ketosis**	**Effect on ventilatory and pulmonary function parameters**
Rubini et al. (29)	32 healthy subjects	ketogenic diet (<30 g CHO/day, 848 Kcal) with phytoextracts, followed by low-carbohydrate no- ketogenic diet (80 g CHO/day, 938 Kcal) with phytoextracts, followed by Mediterranean diet (210 g CHO/day, 1,400 Kcal)	20 days of ketogenic diet, 20 days of low-carbohydrate non-ketogenic diet, 2 months of Mediterranean diet	Yes	- Reduced carbon dioxide end-tidal partial pressure - No significant change in oxygen uptake, carbon dioxide production, nor expired total ventilation
Kwan et al. (30)	6 healthy female subjects	low-carbohydrate diet (<50 g CHO/day), isoenergetic with the usual diet of each subject	1 week	Yes	- Reduced pressure of expired carbon dioxide; trend for reduction in carbon dioxide production - Peak expiratory flow rate and functional residual capacity increased respect to the baseline - No change in resting ventilation and breathing frequency
Angelillo et al. (31)	14 patients with COPD and chronic hypercapnia	liquid diets; low-carbohydrate high-fat (28% calories from CHO, 55% from fat), moderate-carbohydrate moderate-fat (53% calories from CHO and 30% from fat) and high-carbohydrate low-fat (74% calories from CHO, 9.4% from fat); caloric intake tailored on each patient's requirement	5 days for each diet, sequence of diets assigned randomly	No	- Lower CO_2_ production and lower arterial pCO_2_ with the low-carbohydrate diet - No differences in oxygen consumption - Amelioration of forced vital capacity and forced expiratory volume in 1 second (FEV_1_) observed at the end of the administration of all diets - No change in respiratory frequency; trend for reduction in minute ventilation with the low-CHO diet
Tirlapur et al. (32)	8 clinically stable COPD patients with chronic hypercapnic respiratory failure; six obese and twi non-obese	Low-calorie low-carbohydrate diet (30 g CHO/day, 600 Kcal/day)	2–8 weeks	Not specifically evaluated	- Increased arterial oxygen tension and oxygen saturation - Reduced arterial carbon dioxide tension - Increased one-second forced expiratory volume and forced vital capacity - Reduction of nocturnal hypoxemic dips and apnoeic episodes
Kwan et al. (33)	8 clinically stable COPD patients with chronic hypercapnic respiratory failure; non-obese	2 diets isocaloric to the patients' usual diet (about 2,100 Kcal/day), and each containing 200 or 50 g of CHO/day; control diet with CHO intake around 280 g/day	1 week for each diet	No	- Both diets increased arterial oxygen tension and decreased arterial carbon dioxide tension respect to the control diet - The 50 g CHO diet compared to the 200 g CHO diet further reduced the arterial carbon dioxide tension - Carbon dioxide production and oxygen consumption did not change - No significant changes in pulmonary function tests - Amelioration of dyspnoea during the 50 g CHO diet

*KD, Ketogenic Diet; CHO, carbohydrates; COPD, Chronic Obstructive Pulmonary Disease; CO_2_, carbon dioxide*.

Rubini et al. (29) observed that a ketogenic diet (CHO < 30 g/day) administered for 20 days reduced PETCO_2_ in 32 healthy subjects, without modifications in oxygen uptake, carbon dioxide production nor expired total ventilation, which may be related to a reduction in CO_2_ body stores. Therefore, it may be beneficial for patients with high carbon dioxide arterial partial pressure due to respiratory insufficiency, because it lowers CO_2_ levels without increasing respiratory muscle fatigue, with the consequent risk of respiratory failure on mechanical basis. Interestingly, the reduction of PETCO_2_ was maintained even after the end of the diets, suggesting a long-term effect.

Similarly, Kwan et al. (30) observed that a ketogenic diet (50 g CHO/day) administered for 1 week to 6 healthy female subjects reduced arterial carbon dioxide tension, while resting ventilation and breathing frequency remained unchanged. Interestingly, pulmonary function tests showed an increase in peak expiratory flow rate and functional residual capacity respect to the baseline, respectively, by 5 and 10%.

Sue et al. (44) studied the effects of altering the composition of dietary fat and carbohydrate content on gas exchange, at rest and during exercise, on 8 healthy volunteers. At rest, the mean oxygen uptake did not differ, as did not the minute ventilation, while mean CO_2_ output was significantly less on the low-carbohydrate high-fat diet (10% of calories from CHO), compared to the high-carbohydrate diet (70% of calories from CHO). This differences was smaller during exercise, probably because of the preferential use of glycogen stores from the muscle (44).

On the other hand, one study reported negative effects of a ketogenic diet. The administration of a low-carbohydrate high-fat ketogenic diet, providing 2,400 Kcal/day for 4 weeks in 17 healthy women (<25 g of CHO/day), was associated with an earlier muscle fatigue at higher intensities and during daily life activities, probably because of the reduced glycogen stores in the muscle (45). However, a study on 7 well-trained male cyclists consuming a low-carbohydrate high-fat diet (15–82 g of CHO/day), for a long-time (at least the previous 8 months), showed that these athletes had a similar gluconeogenesis rate and reduced glycogenolysis respect to the 7 athletes fed with a mixed diet (46). These findings suggest that after a long-term adaptation to a low-carbohydrate high-fat diet, liver glycogen contributes to endogenous glucose production during exercise, and that glucose may be preferentially obtained from glycerol derived from lipolysis of intramuscular triglycerides (47), configuring an hypothetical difference among about subjects accustomed to a low-carbohydrate high-fat diet and “newbies.”

## Low-Carbohydrate High-Fat Diets and Respiratory Function in Chronic Obstructive Pulmonary Disease Patients

Many Chronic Obstructive Pulmonary Disease (COPD) patients experience hypercapnia and hypoxemia, therefore a nutritional approach that decreases carbon dioxide production, and consequently respiratory muscles work, is extremely interesting.

Some authors observed an improvement of ventilatory measurements in COPD hypercapnic patients after a low-carbohydrate high-fat diet. In a small sample of 14 patients with chronic hypercapnia and COPD the administration of a low-carbohydrate diet (28% CHO) for 5 days, determined a trend of lower CO_2_ production, lower arterial pCO_2_, respect to moderate-carbohydrate moderate-fat diet and high-carbohydrate low-fat diet. An amelioration of forced vital capacity and forced expiratory volume in 1 s (FEV_1_) were observed for all diets, which may reflect an increase in muscle strength (31). However, some recent papers observed that a KD did not improve muscle strength in healthy women (45) and in trained athletes (48, 49), so we may hypothesize that the amelioration in FEV_1_, which was observed for all diets, was to be ascribed to the beneficial effects of the nutritional support rather than its composition. Also, we may speculate that the effect of the diet on muscle strength would be more evident in non-trained patients than in trained athletes.

Some authors studied the short-term alterations following the administration of a particular meal or supplement. Akrabawi et al. (38) enrolled 36 outpatients with COPD and administered a high-fat meal (55% calories from fat, 25 g of CHO) or a moderate fat meal (41% calories from fat, 35 g of CHO) and observed a higher CO_2_ production and O_2_ consumption in patients fed the moderate-fat meal in the early post-prandial time, probably reflecting the earlier absorption of the meal, and no difference for pulmonary function. A recent study on 60 low-body weight patients with COPD and elevated arterial carbon dioxide tension observed that the group administered a low-carbohydrate high-fat evening supplement with 50–75 g of CHO daily for 3 weeks had an overall improvement in ventilatory status, with decrease in VCO_2_, PaCO_2_, VO_2_ and minute ventilation, and an increase of PaO_2_ and FEV_1_, respect to the group administered a traditional high-carbohydrate diet (60–70% CHO) (50). This confirms the importance of nutritional therapy in malnourished COPD patients, since it has a strong impact on respiratory muscle weakness, and the importance of its macronutrient composition.

Many studies on the effects of carbohydrate loads on respiratory parameters and physical performance in chronic obstructive lung disease patients report a detrimental effect of CHO. High-carbohydrate diets may result in increased CO_2_ production and O_2_ consumption in clinically stable COPD patients (39). Kuo et al. (39) studied 12 clinically stable COPD patients and 12 healthy controls after administering an isocaloric high-fat (55.2% fat and 28.1% CHO) or high-carbohydrate (31.5% fat and 54.5% CHO) liquid meal, and found that the high-fat diet had a small effect on gas exchange parameters and ventilation, while the high-carbohydrates diet resulted in a great increase of CO_2_ production, O_2_ consumption and minute ventilation in COPD patients. Efthimiou et al. (40) studied a small sample of 10 clinically stable patients with the 6 min walking test. They observed that the group administered CHO-rich drink experienced a reduced physical performance correlated to the increased CO_2_ production and a perceived effort to breathe. Similar detrimental effects of CHO on walking and exercise performance were obtained in 18 patients with chronic airflow obstruction (41, 42). Increased CO_2_ production and increased minute ventilation after a high-carbohydrate formula, administered for nighttime enteral feeding, were observed in 10 young adult patients with cystic fibrosis with moderate to advanced lung disease (51). On the contrary, a small study with 13 patients affected by stable airways disease fed a high-carbohydrate meal (480 ml of grape juice and three-fourths cup of sucrose) showed that 7 patients who retained carbon dioxide had an increase in PaO_2_, probably reflecting the increased alveolar ventilation, and had not a significant increase in PaCO_2_, therefore the authors conclude that most patients with chronic airways disease are able to tolerate the increased endogenous carbon dioxide load resulting from a meal high in carbohydrates (52).

## Low-Carbohydrate High-Fat Diets, Respiratory Failure and Mechanical Ventilation

Most patients with COPD and acute respiratory failure have marked reduction of body protein stores (53) and lower muscle concentrations of adenosine triphosphate and creatinine phosphate, and these factors may be an important determinant of respiratory failure (54). Moreover, critically ill patients are more responsive to changes in dietary composition than less critical ones (55).

Some authors suggest that a low-carbohydrate diet may be an effective tool to ameliorate respiratory failure (30, 32, 33).

A study on 8 clinically stable chronic hypercapnic respiratory failure patients with congestive heart failure showed that a very low-calorie ketogenic diet (600 Kcal/day, 30 g of CHO/day), administered for on average 1 month, was effective in ameliorating arterial oxygen tension and oxygen saturation, reducing arterial carbon dioxide tension, and ameliorating electrocardiographic abnormalities associated with hypoxiemia (32). These results were probably to be ascribed to the combined effect of diet composition and weight loss, and even two non-obese patients had beneficial effects from the diet.

Another study on 8 chronic hypercapnic respiratory failure patients administered 200 or 50 g of CHO daily for a week within an isocaloric diet, observed that the reduction in the CHO intake on both diets improved the general well-being of the patients, increased arterial oxygen tension and decreased arterial carbon dioxide tension. The 50 g CHO diet compared to the 200 g CHO diet further reduced the arterial carbon dioxide tension, suggesting that such a diet may be used in patients with intractable respiratory failure (33).

Regarding mechanically ventilated patients, in the literature there are some evidences of a beneficial effect of a low-carbohydrate high-fat diet (55–57). A study on 20 clinically stable ventilated patients showed that low-carbohydrate high-fat enteral feeding (55.2% fat, 28.1% carbohydrate) is able to reduce PaCO_2_ levels and the time of mechanical ventilation (55). A recent study on 100 patients with type II respiratory failure, secondary to pulmonary disease requiring mechanical ventilation, showed a great improvement in arterial carbon dioxide tension and minute volume at weaning with the low-carbohydrate high-fat feeding (55.2% fat, 28.1% carbohydrate) (56). Interestingly, similarly to Al-Saady (55), this was highly significantly associated with less time spent on mechanical ventilation (56). On the other hand, a study on 32 patients requiring mechanical ventilation reported no beneficial effects of a similar low-carbohydrate high-fat enteral nutrition (55.2% fat, 28.1% carbohydrate) in PaCO_2_ levels during weaning from the ventilator, respect to a standard enteral nutrition (30% fat, 53.3% carbohydrate) (58). Another recent study on 51 critically ill ventilated children with pulmonary disease observed that the low-carbohydrate high-fat diet (30% carbohydrate, 50% fat) was effective in reducing carbon dioxide tension but did not reduce the duration or level of ventilatory support (59).

Similarly to healthy subjects and respiratory patients in outpatients conditions, the acute carbohydrate loading has detrimental effects on patients with acute respiratory distress (60, 61) and acute respiratory failure (43, 62, 63), acting as a precipitating factor.

Acute respiratory failure was reported in 3 patients under ventilatory support within hours after the beginning of total parenteral nutrition, probably due its high carbohydrate content. The substantial increase of the carbon dioxide production in these patients unable to increase their ventilatory response, led to development of hypercapnia and respiratory acidosis (43).

## Conclusion and Future Perspective

Ketogenic diet should be considered in severely obese people as a preventive measure for SARS-CoV-2 infection, in order to achieve a fast weight loss preserving lean mass. In addition, KD use may be hypothesized as an adjuvant therapy in obese infected patients.

Data obtained in respiratory patients, mainly lean, are indicative of the safety of low-carbohydrate high-fat diets in respiratory compromised patients, and some beneficial effects on respiratory parameters were recorded. KD administration may be helpful for obese patients with chronic hypercapnia, thanks to the reduced CO_2_ production induced by the diet. Many respiratory patients are malnourished, and obese patients themselves are frequently sarcopenic, therefore an adequate protein supplementation is mandatory, since malnutrition may worsen the general prognosis. Many supplements for malnourished patients are rich in carbohydrates, and this is detrimental for their respiratory function, as extensively seen above. KD may be useful for obtaining an adequate protein intake, reducing the ventilatory requirements, the dyspnea and the risk of muscle fatigue. Anyhow, the studies are pretty old, diet administration quite short, samples relatively small, and ketosis not always addressed. In addition, we focused on chronic respiratory diseases, and not on acute infective respiratory illnesses, which may present other issues. Also active infections, in fact, are considered among the contraindications for VLCKD.

Anyway, KD has anti-inflammatory effects and may reduce the risk of cytokine storm, thanks to the anti-inflammatory effects of ketone bodies, and may have a direct anti-viral effect. Therefore, ketogenic diet may be an effective adjuvant therapy in obese non-critically ill COVID-19 patients, and may even be considered in respiratory lean patients. New focused clinical trials with adequate sample sizes, led by a multidisciplinary experienced team of pneumologists, endocrinologists and nutritionists, are needed to confirm the safety and the beneficial effects on ventilatory parameters of such approach in respiratory patients.

## Author Contributions

EG, LG, and CL: conceptualization. EG and RT: writing—original draft preparation. SM, AL, LG, and CL: writing—review and editing. All authors have read and agreed to the published version of the manuscript.

## Conflict of Interest

The authors declare that the research was conducted in the absence of any commercial or financial relationships that could be construed as a potential conflict of interest.

## Publisher's Note

All claims expressed in this article are solely those of the authors and do not necessarily represent those of their affiliated organizations, or those of the publisher, the editors and the reviewers. Any product that may be evaluated in this article, or claim that may be made by its manufacturer, is not guaranteed or endorsed by the publisher.
